# A Serous Cystic Tumor and a Pancreatic Neuroendocrine Tumor in the Same Patient: A Case Report and Literature Review

**DOI:** 10.7759/cureus.61159

**Published:** 2024-05-27

**Authors:** Alexander Aguilar, Vanessa García Gómez, Santiago Ortiz, Sara Vélez Garcés

**Affiliations:** 1 Radiology, Universidad Pontificia Bolivariana - Cedimed, Medellin, COL; 2 Radiology, Hospital Pablo Tobón Uribe, Medellin, COL; 3 Radiology, Universidad Pontificia Bolivariana, Medellin, COL

**Keywords:** pancreatic cyst, magnetic resonance imaging, serous neoplasms, pancreatic neoplasms, neuroendocrine tumors

## Abstract

A serous cystic tumor is a rare entity that has a benign course. Its imaging characteristics, such as the presence of multiple cysts with or without nodular enhancement, can simulate other cystic or solid lesions of the pancreas. Identification of the enhancing scar with punctate calcifications on computed tomography (CT) or magnetic resonance imaging (MRI) may be a distinctive finding suggesting this diagnosis. Neuroendocrine tumors of the pancreas are a different and also rare entity. In images, they have early arterial enhancement. In MRI, they are hyperintense on T2 and hypointense on T1, with avid contrast enhancement. A case of a patient with two focal lesions in the pancreas is presented and the importance of integrating clinical findings, semiology in diagnostic images and, if applicable, the histopathological result for the optimal management of pancreatic tumors is illustrated, highlighting the crucial role of a radiologist in this process.

## Introduction

Pancreatic tumors are lesions that, with the increase in the use of diagnostic imaging, have had an increase in prevalence. It is the radiologist's duty to know and distinguish pancreatic lesions, helping the clinician or surgeon to make the best decision in the treatment [1]. Here, the case of a patient with two pancreatic lesions of synchronous presentation with different histological origins is presented.

## Case presentation

**Figure 1:**
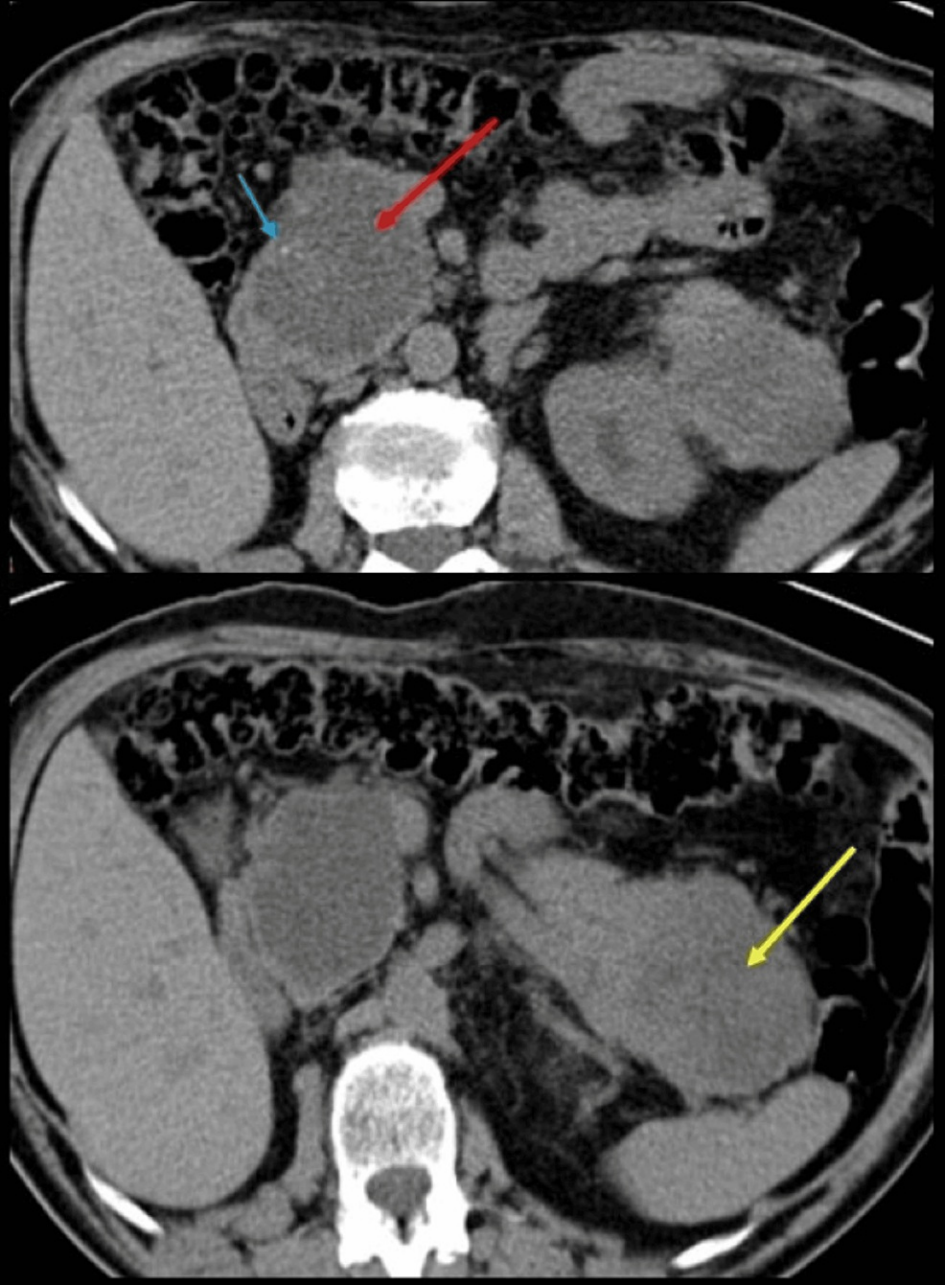
Abdominal tomography without contrast (uroCT). In the head of the pancreas, a rounded, hypodense lesion (red arrow) with small calcifications (blue arrow) is observed without dilating or contacting the main pancreatic duct. The second lesion located on the tail (yellow arrow) is more hyperdense than the one on the head.

The patient was a 91-year-old male. He had a history of hypertension and chronic kidney disease. In urology controls, he had deterioration in his kidney function. Abdominal tomography without contrast (uroCT) was requested; two pancreatic lesions were found, one in the head and the second in the tail with different imaging behavior (Figure 1). It was decided to characterize the lesions by MRI.

**Figure 2:**
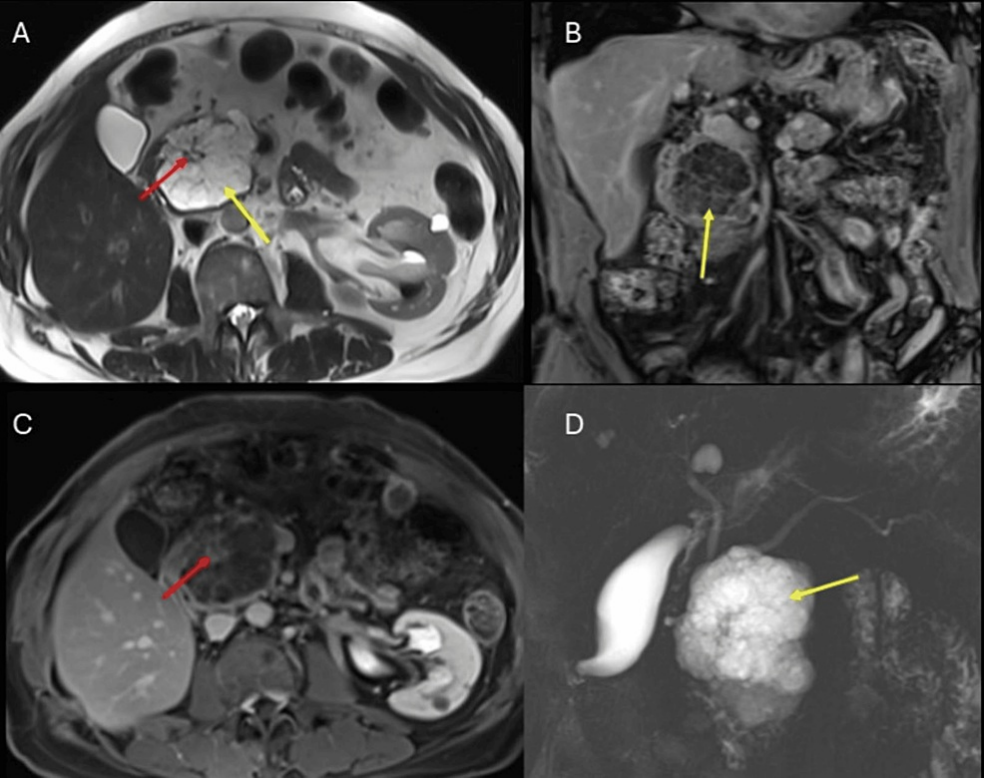
Serous cystic tumor of the pancreas. Contrast-enhanced magnetic resonance imaging of the abdomen. A: T2-weighted sequence in the axial section; B: T1-weighted sequence after gadolinium administration in the coronal section; C: T1-weighted sequence after gadolinium administration in the axial section. D: Cholangioresonance sequence. A lesion is observed in the head of the pancreas, made up of multiple confluent cysts (yellow arrows) and thin septa inside that converge in a central scar and present enhancement with the contrast medium (red arrows). Findings compatible with a serous cystic tumor of the pancreas.

The head lesion is rounded, made up of multiple confluent cysts and thin septa inside, it is hyperintense on T2 and hypointense on T1, with facilitated diffusion; after the administration of gadolinium, the lesion does not enhance and the thin septa have enhancement that converge in a center (scar) (Figure 2).

**Figure 3:**
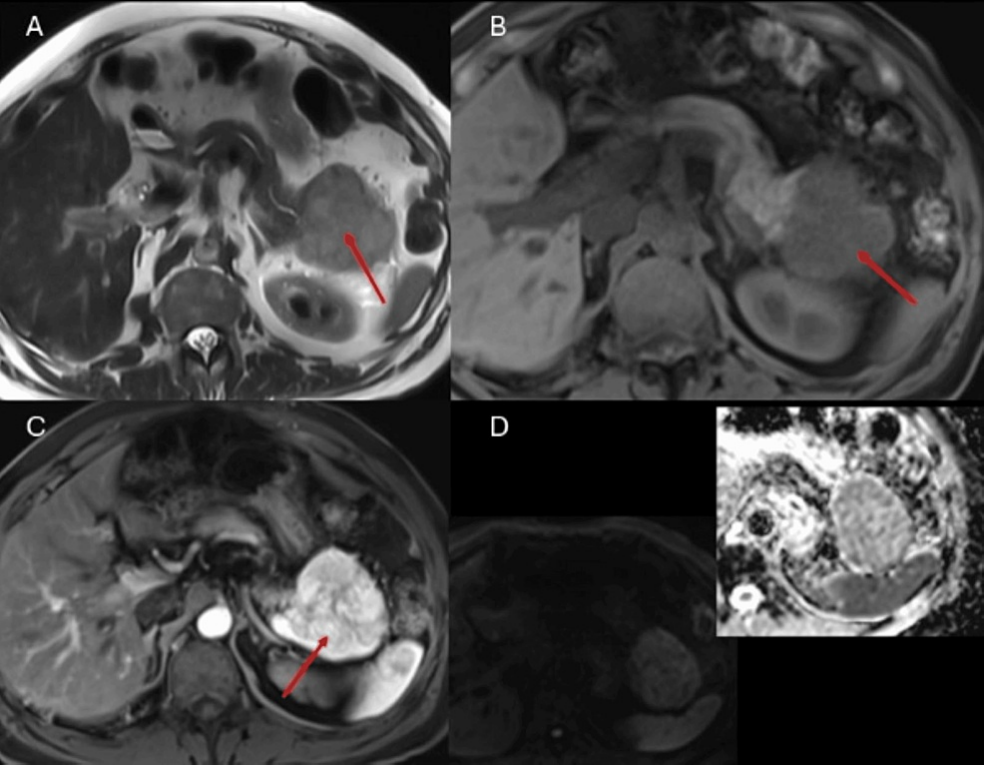
Pancreatic neuroendocrine tumor. Contrast-enhanced magnetic resonance imaging of the abdomen. A: T2-weighted sequence in axial section; B: T1-weighted sequence without contrast in axial section; C: T1-weighted sequence after gadolinium administration in axial section. D: diffusion sequences. A solid, rounded lesion is observed, located in the tail of the pancreas, it is hypointense on the T1 and T2 sequences with respect to the pancreatic parenchyma (red arrows) and has mild diffusion restriction. Findings compatible with pancreatic neuroendocrine tumor.

The tail lesion is a well-defined, solid lesion, hypointense with respect to the pancreatic parenchyma with avid and homogeneous enhancement and slight restriction to diffusion (Figure 3). Due to its imaging characteristics, a serous cystic tumor was suggested as the first possibility for the head lesion and a neuroendocrine tumor (NET) for the tail lesion.

**Figure 4:**
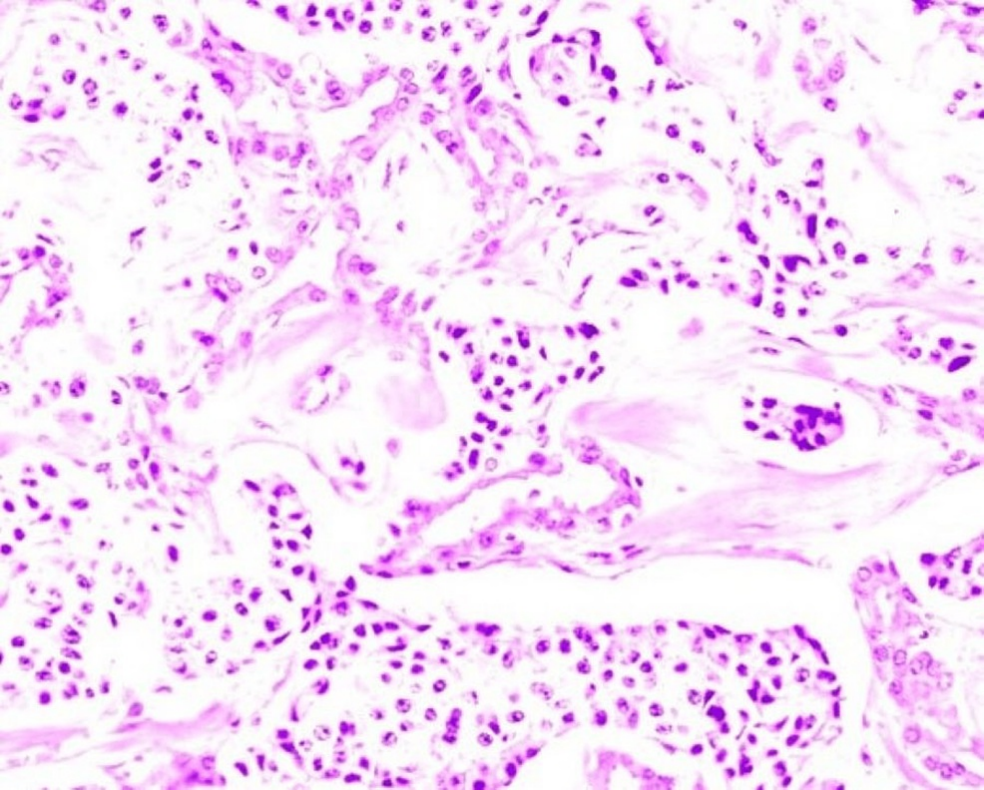
Pancreatic tail tumor histology. Groups of rounded and oval cells made up of eosinophilic cytoplasm. Immunohistochemistry was performed with positivity for chromogranin, synaptiphysin, CD56, cytokeratin cocktail, and CA19-9. The findings confirm a grade 1/3 well-differentiated neuroendocrine tumor.

He was taken to ultrasound endoscopy and biopsy for the tail lesion where a grade one pancreatic NET was confirmed (Figure 4). Due to its low probability of malignancy, it was decided to follow up. The head tumor was not punctured due to its classic characteristics of a serous microcystic tumor.

**Figure 5:**
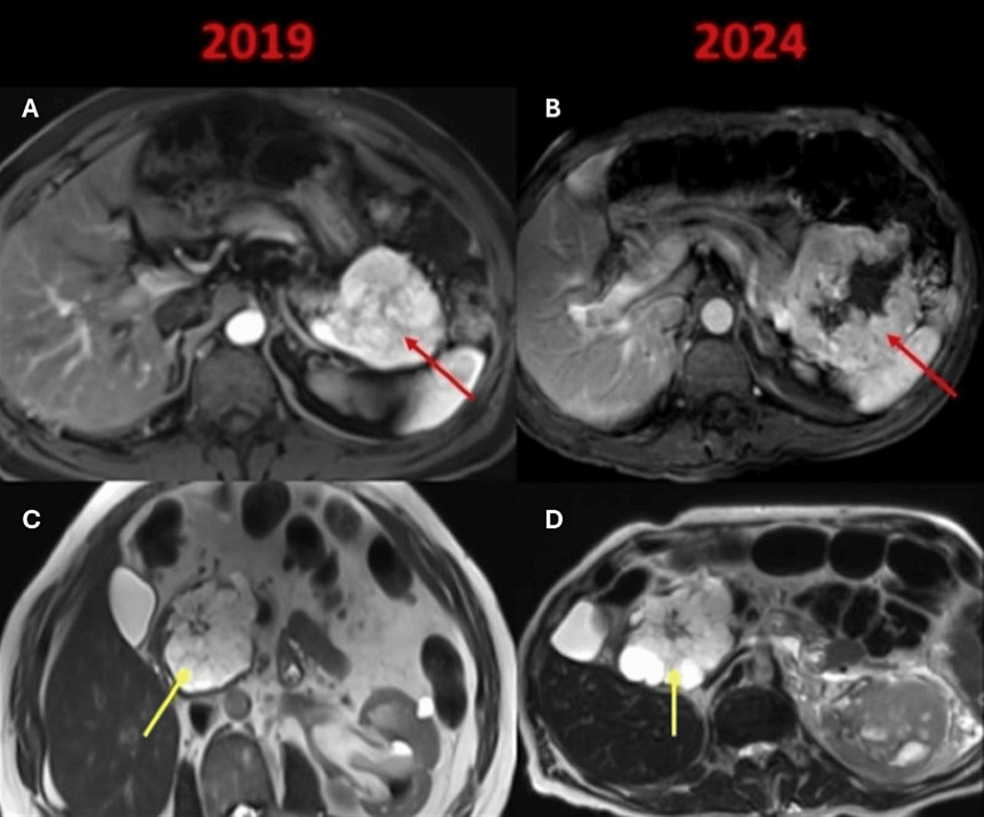
Comparative MRI between diagnosis and five years later. Comparison of imaging follow-up is made five years later. A and B: The neuroendocrine tumor in the tail of the pancreas presents an increase in size at the expense of the cystic degeneration that these tumors can suffer (red arrows). C and D: The lesion of the pancreatic head has no significant changes (yellow arrows), which confirms the benignity of the lesion (serous cystic tumor).

The patient did not present any clinical symptoms and presented five years later for review. A new MRI was performed where growth of the NET was observed at the expense of central cystic degeneration (Figure 5); there is no distant metastasis or vascular compromise, while the serous cystic tumor does not present significant changes, which confirms the benign nature of the lesion.

## Discussion

Serous cystic tumor

A serous cystic tumor of the pancreas is a rare tumor lesion, corresponding to at least 1% of the pathologies that affect the organ [1].

Its behavior is generally benign. It can resemble other benign or malignant tumors [2]. It affects women more than men in a ratio of 2.5:1 and typically presents in the seventh decade of life. An increase in prevalence has also been seen in people with Von Hippel-Lindau (VHL) disease [3].

Up to 80% of these tumors are asymptomatic; the largest ones can cause symptoms such as abdominal pain, dyspepsia, nausea, vomiting, jaundice, and sensation of abdominal mass [3]. Diagnostic images have a very important role in the diagnosis and monitoring of these tumors. Within the imaging findings, two main patterns can be observed: the polycystic pattern which is characterized by the appearance of multiple small cysts (more than six and less than 2 cm), and the second is the honeycomb pattern where multiple cysts come together which simulates an enhancing mass on CT or MRI [4]. A finding that is considered pathognomonic is the presence of an enhancing scar with punctate calcifications that appears in up to 30% [5].

A serous cystic tumor can be diagnosed by imaging with very high specificity. The main differential diagnoses are cystic NETs and intraductal papillary mucinous neoplasm. Asymptomatic patients do not require follow-up imaging. Surgery is considered when the patient has gastrointestinal symptoms caused by the tumor [5].

Pancreatic neuroendocrine tumor

Pancreatic NETs are rare and can appear sporadically (up to 80%) or be associated with diseases such as multiple endocrine neoplasia type 1, neurofibromatosis, and VHL disease [6].

They can be divided into two types, functioning and non-functioning. The former present a clinical picture that depends on the type of hormone they produce and the non-functioning ones generally cause the mass effect [7].

Diagnostic images play a fundamental role in diagnosis and follow-up. In CT, pancreatic NETs and associated metastases enhance significantly in the arterial phase and persist with high attenuation during the portal and late phase. They are generally small lesions [8]. 

Larger lesions can undergo changes over time or grow due to cystic degeneration, necrosis or calcifications, as in our patient who had cystic degeneration (Figure 5).

In MRI, a lesion will typically be found to be hypointense on T1, hyperintense on T2, and during the portal and late phases, isointense compared to the adjacent pancreatic tissue [9]. These lesions require follow-up with MRI and PET-CT. In most cases, surgical treatment is preferred [9]. None of these lesions dilate the pancreatic duct in general.

The only association between the two tumors described in the literature is VHL syndrome [2]. However, in our patient, that pathology was not clinically suspected.

## Conclusions

Pancreatic tumors will always be a diagnostic challenge for the radiologist. Due to the greater use of diagnostic images, the prevalence of pancreatic lesions has increased. For this reason, the radiologist must know the main cystic and solid lesions of the pancreas and their imaging characteristics and thus help the treating physician make an accurate therapeutic decision depending on the suspected tumor.
